# Four-Dimensional (4D) Volume-Based Bolus-Tracking Monitoring for Post-endovascular Aneurysm Repair CT: Feasibility of Combined Hemodynamic and Morphological Imaging

**DOI:** 10.7759/cureus.112050

**Published:** 2026-07-04

**Authors:** Satoshi Takita, Noriyuki Nagami, Reika Oka, Hisanori Shibahara, Osamu Togao

**Affiliations:** 1 Radiology, Saga University Hospital, Saga, JPN; 2 Faculty of Medicine, Saga University, Saga, JPN

**Keywords:** 4d-cta, aortic attenuation, bolus tracking, cardiac chamber attenuation, cta, descending aortic regions of interest, endoleak, evar

## Abstract

Purpose

The purpose of this study was to evaluate the feasibility of a four-dimensional (4D) bolus tracking (BT) workflow for post-endovascular aortic repair (EVAR) aortic CT that combines volume-based 4D monitoring with high-resolution CT angiography (CTA) in a single examination.

Methods

We retrospectively analyzed 35 patients who underwent EVAR and 4D-BT-guided aortic CTA. The 160-mm dynamic volume monitoring acquisition was initiated 15 seconds after contrast injection, and the main helical CTA was triggered when the descending aortic regions of interest (ROI) exceeded 400 Hounsfield Units (HU) (switching time, 7-8 seconds). We assessed arterial CT attenuation at six sites, cardiac chamber attenuation for timing assessment, and radiation dose (CT dose index (CTDIvol)/dose length product (DLP)).

Results

The mean aortic attenuation exceeded 350 HU at all six measurement sites in all cases, with no significant differences among the locations. Cardiac chamber attenuation increased from the right atrium (175 HU) to the pulmonary artery (220 HU) and left atrium (305 HU), supporting consistent arterial phase timing. The mean CTDIvol/DLP was 3.32 mGy/53.2 mGy·cm for volume-based 4D monitoring and 3.2 mGy/255 mGy·cm for CTA; the time-enhancement curve (TEC) peak attenuation exceeded 800 HU in two cases.

Conclusion

In this feasibility study, the 4D-BT workflow provided reproducible arterial enhancement and consistent trigger-based timing while acquiring both 4D-CTA and CTA datasets in a single session. The diagnostic performance for endoleak detection and the net benefit of contrast load and radiation dose require confirmation in comparative prospective studies.

## Introduction

Endovascular aneurysm repair (EVAR) has become the standard treatment for abdominal aortic aneurysms because of its minimally invasive nature and favorable short-term outcomes [1]. However, long-term success depends on careful follow-up, including the detection of endoleaks, which are challenging to detect [2]. Computed tomography (CT) is widely used for post-EVAR surveillance because of its high spatial resolution and ability to detect subtle vascular changes in the aorta [3]. Recently, dynamic imaging techniques, such as four-dimensional (4D) CT angiography (CTA), have received attention as promising methods for evaluating contrast agent flow [4]. Among these, 4D-CTA enables continuous and dynamic visualization of contrast agent flow over time, providing temporal and spatial information [5]. This may improve the detection and characterization of endoleaks by capturing subtle variations in contrast enhancement [6].

Currently, both hemodynamic information within the stent graft and detailed vascular morphology are required for the accurate evaluation of endoleaks [3,4]. Typically, these data are obtained using separate imaging techniques: 4D-CTA for hemodynamic assessment and conventional CTA for morphological evaluation [5]. However, in current clinical practice, acquiring both hemodynamic and morphological data in a single imaging session remains challenging. In conventional protocols, 4D-CTA and CTA are typically performed as separate scans, each requiring its own contrast injection. Consequently, when both datasets are required, additional imaging studies are often necessary, which may increase contrast agent use and radiation exposure [7]. In contrast, the proposed workflow enables both examinations to be performed with a single contrast injection, potentially allowing the total contrast dose to remain comparable to that of a single examination. Moreover, because the 4D monitoring component is intended primarily for determining scan timing and is performed at a low-dose setting, this approach may also help reduce overall radiation exposure compared with performing the examinations separately.

In recent years, advances in CT scanner technology, including increased detector rows and faster scanning speeds, have led to the development of various contrast-enhanced imaging techniques aimed at capturing images at optimal times for specific diagnostic purposes. Currently, common methods for determining the arrival time of contrast agents include bolus tracking (BT) [8] and test injection [9] techniques. All these methods are designed to generate a time-enhancement curve (TEC) at a predefined axial slice for contrast monitoring.

To address the issue of being unable to obtain both hemodynamic and morphological information in a single examination, we proposed a new monitoring technique, the 4D-BT method. This approach leverages the capability of 4D-CTA to perform BT across an entire volume of interest, rather than being limited to a single axial slice. As a result, it enables the simultaneous determination of the optimal timing for contrast-enhanced imaging and the acquisition of both 4D-CTA and conventional CTA datasets. This study aimed to evaluate whether this method enables image acquisition at the optimal time, primarily based on arterial CT attenuation values. Although the radiation dose was not directly evaluated, low-dose scan parameters were implemented as a potential advantage.

## Materials and methods

This was a retrospective study conducted at the Saga University Hospital, Saga City, Japan. The study was approved by the Institutional Review Board of Saga University Hospital (approval number: 2022-06-07).

Study population

The inclusion criteria were consecutive post-EVAR patients during the study period who underwent the first follow-up CT with the 4D-BT protocol. The exclusion criteria were contrast extravasation and failure to meet the predefined contrast injection criteria (e.g., incomplete injection or deviation from the institutional iodine dose protocol).

The study included 35 consecutive post-EVAR patients (35 examinations) who underwent aortic CT using the 4D-BT method at their first post-EVAR follow-up between January and July 2024. All patients had a 22-gauge intravenous catheter placed in the right upper extremity in the CT suite.

Four-dimensional bolus tracking method

**Figure 1:**
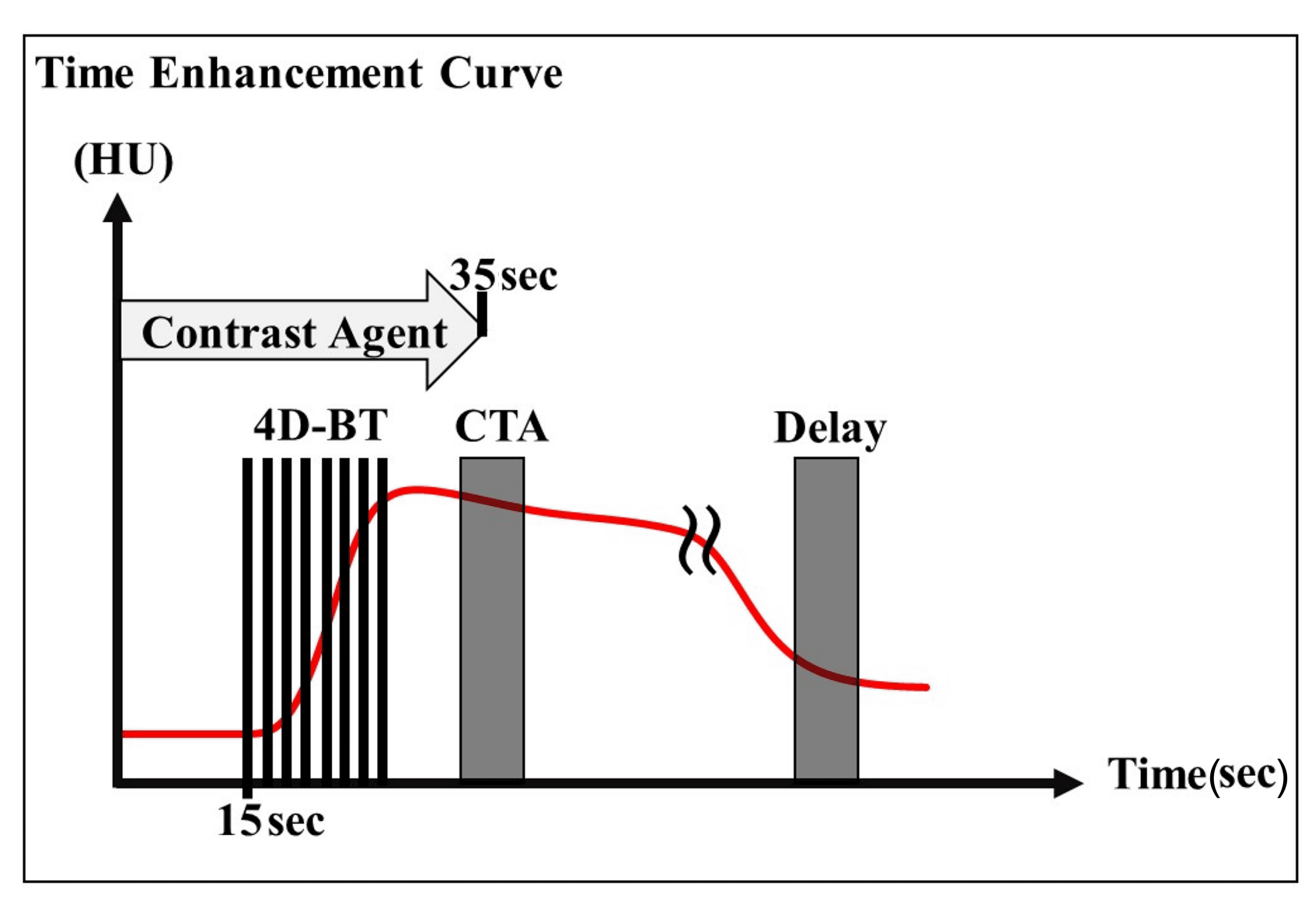
Overview of the 4D-BT workflow: 4D Prep monitors a 160-mm volume and triggers CTA at optimal enhancement. 4D: four-dimensional; BT: bolus tracking; CTA: computed tomography angiography; HU: Hounsfield units The image was created using Microsoft PowerPoint (Microsoft Corporation, Redmond, Washington, United States)

Figure 1 shows an overview of the 4D-BT method used in this study. The 4D-BT method incorporates dynamic volume (Dy-Volume) monitoring, which involves repeated volumetric acquisitions covering a 160 mm z-axis range, into the monitoring phase of the conventional BT method. This workflow is based on Real Prep (Canon Medical Systems Corporation), which monitors contrast dynamics and automatically detects contrast arrival in the target vessel to trigger the image acquisition.

In this study, to distinguish it from conventional slice-based monitoring in the BT method, we used a 160-mm Dy-volume acquisition for bolus-tracking monitoring and 4D data collection (hereafter, volume-based 4D monitoring). Other scanning parameters and contrast protocols were essentially the same as those used for the standard BT method.

Volume-based 4D monitoring acquisition was initiated 15 seconds after contrast injection. When the CT attenuation within the regions of interest (ROI) placed in the descending aorta reached the trigger threshold (400 HU), the system initiated the transition to helical scanning for the aortic CTA. The switching process, including the breath-hold announcement, took approximately 7-8 seconds to complete. This delay represented the fixed transition time after triggering and was consistent across all cases. In patients with reduced cardiac output, the monitoring duration was prolonged rather than the post-trigger delay time.

At our institution, the 4D-BT protocol was used as the first post-EVAR follow-up CT examination. Pretest suspicion of an endoleak, when present, was determined based on routine clinical assessment and any available prior imaging findings; therefore, not all patients were necessarily classified as “suspected endoleak” cases before the 4D-BT scan. The monitoring volume was positioned to include the stent graft region, and the target trigger CT value was set at 400 HU. The transition from volume-based 4D monitoring to the main aortic CTA was performed manually in all cases (100%); accordingly, no separate subgroup analysis based on the transition type was performed. When the trigger threshold was reached earlier than expected, helical CTA was initiated according to the protocol, even before the completion of contrast injection.

All examinations were performed using a 320-row CT scanner (Aquilion ONE INSIGHT Edition; Canon Medical Systems Corporation, Ōtawara, Tochigi, Japan) for both monitoring and main CTA scans.

Scan parameter

The scan parameters were as follows: For volume-based 4D monitoring, the tube voltage was 70 kV, and the tube current was fixed at 150 mA, with a gantry rotation time of 0.24 seconds and a scan interval of 1.5 seconds. For the main CTA scan, the tube voltage was 100 kV, and automatic exposure control (AEC) was used with a target image noise level corresponding to an SD of 15 HU at a 5-mm reference reconstruction. The gantry rotation time was 0.24 s, and the pitch was 1.388. The average CT dose index (CTDIVol)/dose length product (DLP) was approximately 3.32 mGy/53.2 mGy·cm for volume-based 4D monitoring and 3.2 mGy/255 mGy·cm for the CTA.

Image reconstruction

Volume-based 4D monitoring images were reconstructed using deep learning-based noise-reduction reconstruction with a high-resolution body kernel (Precise IQ Engine (PIQE), Body L3; Canon Medical Systems Corporation) at a slice thickness of 1.0 mm and a 512 × 512 matrix. The CTA images were reconstructed using deep learning-based reconstruction (Advanced Intelligent Clear-IQ Engine (AiCE), L2; Canon Medical Systems Corporation) with a slice thickness of 1.0 mm for the entire aorta. For the stent graft region, images were additionally reconstructed using PIQE (Body L2) at a slice thickness of 0.5 mm with a 1024 × 1024 pixel matrix.

Contrast agent protocol

The amount of contrast agent administered was 480 mgI/kg, according to institutional standards, and this iodine dose was applied uniformly to all patients. To provide sufficient time for 4D acquisition, the injection duration was set to 35 seconds. A variable injection technique (with an injection rate variation factor of 0.5) was used to enhance peripheral vascular enhancement. Furthermore, to maintain a stable injection rate, the iodine concentration of the contrast agent was adjusted according to body weight, as illustrated in Table 1.

**Table 1:** Body-weight–based protocol for contrast agent concentration and injection settings.

Body Weight（kg）	Concentration（mgI/mL）	Syringe（mL）
< 50	240	100
51～65	300	100
66～74	350	100
> 75	370	100

Evaluation of the proposed method

Quantitative Assessment of Aortic Enhancement in CTA

To assess the distribution and uniformity of contrast enhancement in the aorta during 4D-BT-guided CTA, the mean CT attenuation values (in HU) were measured at six anatomical levels: ascending aorta, aortic arch, thoracic aorta (at the level of the carina), abdominal aorta (at the level of the renal arteries), common iliac arteries, and femoral arteries. This evaluation was performed to determine whether adequate and consistent enhancement was achieved across the entire arterial tree during the main scans.

Assessment of Scan Timing Appropriateness Based on Cardiac Circulation

To further evaluate the appropriateness of the scan timing achieved with the 4D-BT method, the average CT attenuation values were measured at key locations in both the right and left cardiac circulation. The measurement sites included the right atrium, pulmonary artery, and left atrium. By comparing the enhancement in these chambers, the timing of contrast passage from venous return to systemic arterial circulation can be indirectly assessed, offering insights into whether the scan was performed during the optimal arterial phase.

Analysis of the TEC From Volume-Based 4D Monitoring

The TEC derived from the volume-based 4D monitoring scan was analyzed to evaluate the dynamic behavior of contrast enhancement within the ROI set in the descending aorta [10]. The TEC provided a visual representation of contrast arrival, peak enhancement, and washout, which was used to validate the triggering accuracy of the 4D-BT method and confirm that image acquisition occurred within the optimal enhancement window.

Statistical analysis

All statistical analyses were performed using EZR (version 1.52; Jichi Medical University, Tochigi, Japan), a graphical user interface for R (R Foundation for Statistical Computing, Vienna, Austria) [11]. Differences in CT attenuation among the three groups were assessed using the Kruskal-Wallis test. For pairwise comparisons between the two groups, the Steel-Dwass method was applied with adjustment for multiple comparisons. A two-sided P value < 0.05 was considered statistically significant.

## Results

A total of 35 post-EVAR patients were included in the study. The characteristics of the cohort are given in Table 2.

**Table 2:** Baseline characteristics of the study cohort (N=35)

Characteristic	Values
Age (years), median (range)	73 (58-99）
Male, n (%)	21 (60%)
Female, n (%)	14 (40%)
Weight (kg), median (range)	58.2 (37.8 - 85）
Injection start rate (ml/sec), mean±SD	3.53 ± 0.21
Contrast agent volume (ml), mean±SD	93.43 ± 5.69

**Figure 2:**
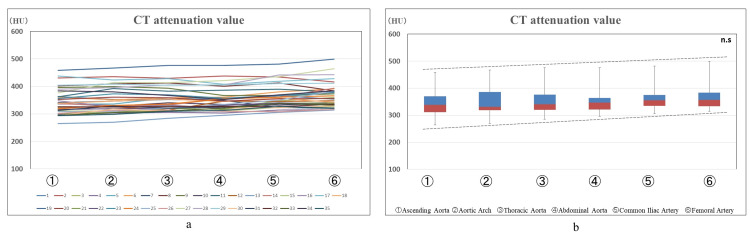
Aortic enhancement across six arterial levels. (a) CT attenuation at the ascending aorta, aortic arch, thoracic aorta (carina level), abdominal aorta (renal-artery level), common iliac arteries, and femoral arteries. (b) Summary of attenuation across the six sites; except for one case, CT attenuation exceeded 350 HU, and no significant differences were observed among the six measurement locations.

The variable injection technique consistently provided a high enhancement in the peripheral arteries, including the femoral and iliac arteries (Figure 2). At the six measurement sites, the median aortic CT attenuation values were 337.91 HU in the ascending aorta, 331.28 HU in the aortic arch, 340.52 HU in the thoracic aorta, 347.08 HU in the abdominal aorta, 355.00 HU in the common iliac arteries, and 356.36 HU in the femoral arteries. Overall, attenuation was relatively consistent across the sites, with higher enhancement observed in the distal arteries (common iliac and femoral arteries). In one patient, CT attenuation from the ascending to the abdominal aorta was lower, with values below 300 HU. This outlier was a low-body-weight patient; therefore, the total contrast volume was smaller under the fixed iodine-dose protocol. Because the injection duration was set to 35 seconds, the injection rate may not have increased sufficiently to achieve a higher peak enhancement, which likely contributed to lower proximal aortic attenuation. Despite this, the images were considered diagnostically acceptable, and the CT attenuation in the peripheral arteries still exceeded 300 HU, indicating that distal enhancement was maintained.

**Figure 3:**
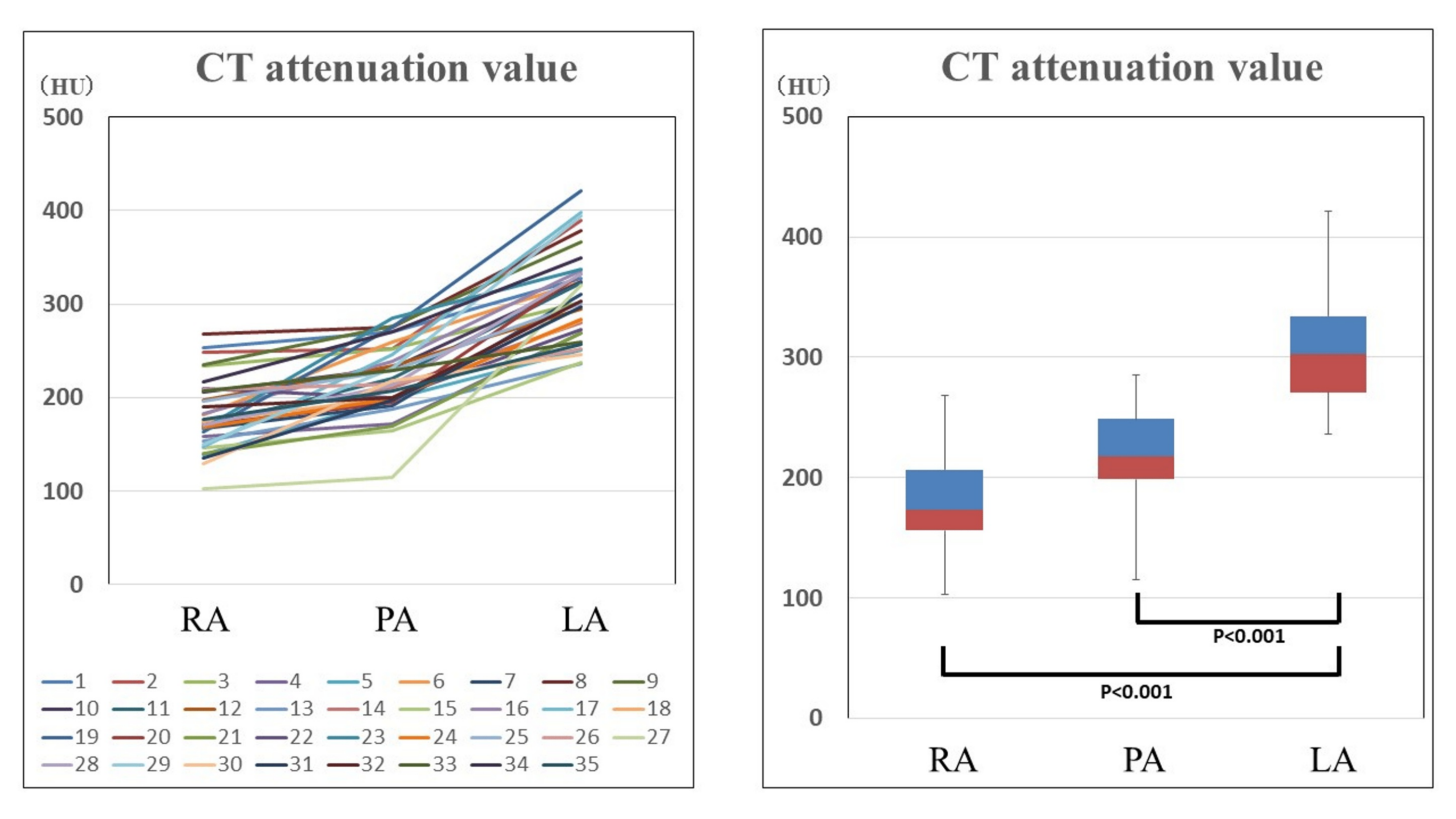
Cardiac timing evaluation. (a) CT attenuation in the RA, PA, and LA. (b) Summary of CT attenuation across the three cardiac locations (RA < PA < LA), supporting arterial-phase acquisition of the main scan. RA: right atrium; LA: left atrium; PA: pulmonary artery

The CT attenuation increased in a stepwise manner from the right atrium to the pulmonary artery and then to the left atrium (Figure 3). The mean CT attenuation values were 175 HU in the right atrium (RA), 220 HU in the pulmonary artery (PA), and 305 HU in the left atrium (LA), reflecting the expected physiological progression of contrast enhancement through the right heart, pulmonary circulation, and into the left atrium. A three-group comparison using the Kruskal-Wallis test showed a significant difference among RA, PA, and LA (Kruskal-Wallis statistic = 181.17, P < 0.001). Post hoc pairwise comparisons using the Steel-Dwass method demonstrated significant differences for all pairs: RA vs. PA (statistic = 4.140, P < 0.001), RA vs. LA (statistic = 7.053, P < 0.001), and PA vs. LA (statistic = 6.372, P < 0.001), with the overall pattern RA < PA < LA. This pattern supports the notion that the main scan was performed during the appropriate arterial phase.

**Figure 4:**
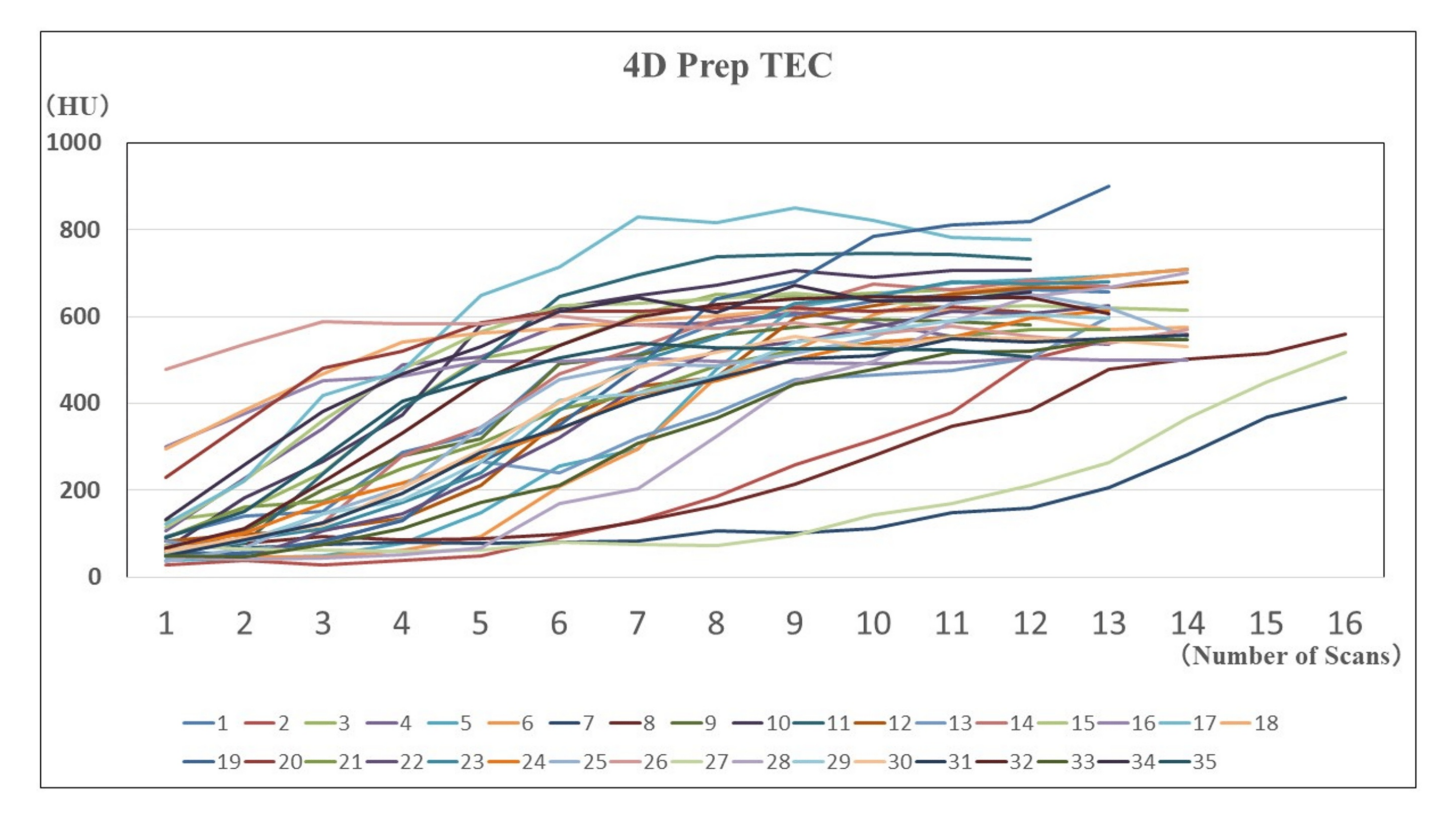
4D prep time-enhancement curve in the descending aorta; peaks > 800 HU in two cases. 4D: four-dimensional; HU: Hounsfield unit; TEC: time-enhancement curve

In all 35 cases, the transition to the main aortic CT scan was triggered after the CT attenuation in the descending aortic ROI exceeded 400 HU. As shown in Figure 4, the TEC peak attenuation exceeded 800 HU in some cases, indicating a strong enhancement during volume-based 4D monitoring.

These findings suggest that the 4D-BT workflow can align CTA acquisition with a highly enhanced arterial phase; however, the diagnostic performance for endoleak detection was not evaluated in this study.

## Discussion

The 4D-BT workflow consistently achieved a mean aortic CT attenuation of >350 HU across all examined cases, suggesting that the contrast injection settings and trigger-based timing were adequate for reproducible arterial enhancement in this cohort. This feasibility study primarily evaluated the enhancement and scan timing using CT attenuation-based indices. Although iodine and radiation dose indices were recorded, we did not assess their clinical impact, perform comparisons with standard surveillance protocols, or evaluate the diagnostic performance for endoleak detection.

**Figure 5:**
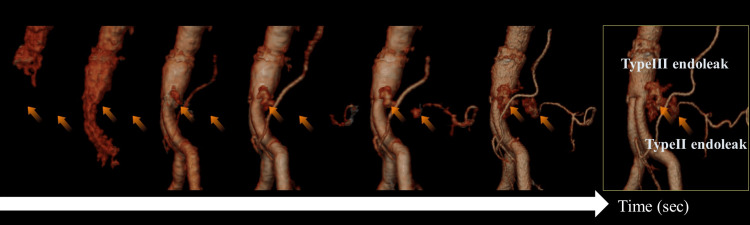
Images illustrating the 4D-BT workflow in a post-EVAR examination, suggesting its potential use for combined hemodynamic and morphological assessment. 4D: four-dimensional; BT: bolus tracking; EVAR: endovascular aneurysm repair Images were processed using the institutional workstation, Ziostation REVORAS (Ziosoft USA, Inc., Newark, California)

The variable injection scheme with a higher initial injection rate and an appropriate iodine dose was used to achieve high attenuation, even in peripheral arteries (Figure 5). This observation raises the hypothesis that the protocol could facilitate the visualization of small feeding vessels related to Type II endoleaks; however, this study did not quantify endoleak detectability or type-specific diagnostic performance.

A steeper rise in the TEC was observed with a higher initial injection rate, which may allow for a shortening of the monitoring duration in some protocols. In the present workflow, scan timing was supported by chamber attenuation patterns: right atrial attenuation was lower than pulmonary arterial and left atrial attenuation, while left atrial attenuation exceeded ~300 HU, consistent with arterial-phase acquisition.

In two cases, TEC peak attenuation exceeded 800 HU; however, no apparent beam-hardening artifacts were observed in the stent-graft region, and the very high attenuation did not appear to compromise diagnostic interpretability. Clinically, such temporal volumetric information is expected to facilitate the assessment of contrast inflow patterns in high-flow endoleaks (e.g., Type I/III) and to improve the opportunity to capture intermittent or posture-dependent leaks that may be missed on single-phase CTA [12-14]; however, these endpoints were not evaluated in the present study and should be confirmed in future studies. These potential advantages should be interpreted as hypotheses, as endoleak detection rates, reference standards, and clinical outcomes were not evaluated.

Overall, this study positions 4D-BT primarily as a feasibility evaluation of the enhancement and timing of a combined 4D-CTA plus CTA workflow in a single session. Future studies should compare this approach with standard surveillance strategies (including multiphase CTA and contrast-enhanced ultrasound) using objective image quality metrics and diagnostic endpoints [15]. In particular, prospective studies should evaluate diagnostic performance against a predefined reference standard (e.g., a composite of multiphase CTA and contrast-enhanced ultrasound, with catheter angiography and/or clinical follow-up as available) and report sensitivity/specificity by endoleak type, along with comparative radiation dose (CTDIvol/DLP) and iodine dose.

This study had several limitations. First, it was a single-center, retrospective study with a limited sample size (n = 35). Consequently, the generalizability of the findings is constrained, necessitating further validation through multicenter and prospective studies to confirm the clinical applicability of the 4D-BT method in this context. Second, the potential image noise caused by respiratory motion or cardiac pulsations was not fully evaluated in this study. Such motion artifacts may influence both the accuracy of the TEC and the stability of dynamic volumetric acquisition. Future studies incorporating motion correction or respiratory gating techniques can further improve the reliability of the data. Third, the strong enhancement achieved in some cases may lead to beam-hardening artifacts, particularly near the high-density graft materials. The optimization of contrast injection parameters and reconstruction algorithms will be important for minimizing these effects in future studies. Despite these limitations, our findings provide valuable insights into the feasibility and diagnostic potential of the 4D-BT method, establishing a foundation for larger-scale studies aimed at improving post-EVAR imaging strategies.

## Conclusions

The 4D-BT workflow enables acquisition of both temporal (4D-CTA) and morphological (CTA) datasets in a single post-EVAR CT examination with consistent trigger-based timing and uniform arterial enhancement. This feasibility study did not directly evaluate the diagnostic performance for endoleak detection or compare iodine load and radiation dose with standard surveillance protocols; therefore, diagnostic superiority should not be inferred from the present results. Clinically, this workflow is expected to be most useful in patients in whom a detailed hemodynamic assessment is desired, such as in cases with suspected endoleaks that are difficult to classify on single-phase CTA, mixed or coexisting endoleak types, and early high-flow endoleaks (e.g., Type I and/or Type III) that require precise characterization. Future prospective studies should assess endoleak detection performance by type using a predefined reference standard and compare iodine and radiation doses with standard protocols.
